# Useful Hints for the Laboratory

**Published:** 1900-03

**Authors:** Arthur E. H. Lister

**Affiliations:** Lincoln, Eng.


ARTICLE III.

### USEFUL HINTS FOR THE LABORATORY.

ARTHUR E. H. LISTER, LINCOLN, ENG.

It is not generally known how to obtain a good die from a lower model that has six or eight anterior teeth standing, and the jaw very much undercut below the necks of the teeth, lingually.

After the model has been prepared in the usual manner, oil or vaseline the parts adjoining the undercut, then flow plaster inside and build up the teeth as high as the plate is intended to go. While still plastic, stick in two pins at a suitable distance apart and press them down until they touch the model. When fully set, you can withdraw pins, and by gently tapping the model, the plaster will leave it clean.

Thoroughly dry plaster, then replace and stick it with a little wax to model; take an impression in sand in the usual way. Remove plaster from model, see that it is dry, carefully replace in sand impression, and either stick the pins through the holes "already made" into the sand, or hold it lightly in place with a knitting needle, or anything suitable, until you pour in the metal.

After a little experience with the above process you will be surprised to find that in nine cases out of ten you can procure a die on which to finish striking up a plate, which you could not have hoped to secure in any other conceivable way, to say nothing of the time saved.

When casting plaster impressions, instead of using oil or soap for the separating fluid, paint them over with a paint made of vermillion and oil, then you will be better able to detect the line of demarkation.

To clean the dirt and wax from teeth before trying them in, rub lightly with a soft rag moistened with methylated spirits.

If a vulcanite piece is to be packed upside down, you can economize time by inverting the impression for the duplicate in one-half of the flask, instead of in the usual way. Then when you wax the piece on to the model it is already flasked in one-half and there is no waiting for the plaster to set before you pour the counterpart.

To remove vulcanite from between the teeth, take a stiff, fine needle, mount it in a small handle or broach holder, sharpen it on two sides and you have a useful little tool for the work.

When using water of-ayr stone to erase scratches, it will greatly facilitate the removal if you occasionally dip it in a little pumice powder, which should be kept in a small vessel close at hand.

To remove scratches out of a very deep palate, if you have not a hub brush, round one end of a cork and fix it on lathe mandrel the same as you would a brush, using the usual polishing paste.

Nail a wine cork conveniently on bench and stud it all over with pins (it is surprising to see how many one will hold), then you will always have some handy for sticking in impressions of teeth, etc.

Paste a piece of sand paper within convenient reach underneath the bench, and you have a handy place to strike your matches.—*Items of Interest*

